# Differences in the post-stroke innate immune response between young and old

**DOI:** 10.1007/s00281-023-00990-8

**Published:** 2023-04-12

**Authors:** Mattia Gallizioli, Maria Arbaizar-Rovirosa, David Brea, Anna M. Planas

**Affiliations:** 1 Department of Neuroscience and Experimental Therapeutics, Instituto de Investigaciones Biomédicas de Barcelona (IIBB), Consejo Superior de Investigaciones Científicas (CSIC), S Rosselló 161, planta 6, 08036 Barcelona, Spain; 2 Institut d’Investigacions Biomèdiques August Pi i Sunyer (IDIBAPS), Rosselló 153, 08036 Barcelona, Spain

**Keywords:** Stroke, Brain ischemia, Aging, Inflammation, Innate immunity

## Abstract

Aging is associated to progressive changes impairing fundamental cellular and tissue functions, and the relationships amongst them through the vascular and immune systems. Aging factors are key to understanding the pathophysiology of stroke since they increase its risk and worsen its functional outcome. Most currently recognised hallmarks of aging are also involved in the cerebral responses to stroke. Notably, age-associated chronic low-grade inflammation is related to innate immune responses highlighted by induction of type-I interferon. The interferon program is prominent in microglia where it interrelates cell damage, danger signals, and phagocytosis with immunometabolic disturbances and inflammation. Microglia engulfment of damaged myelin and cell debris may overwhelm the cellular capacity for waste removal inducing intracellular lipid accumulation. Acute inflammation and interferon-stimulated gene expression are also typical features of acute stroke, where danger signal recognition by microglia trigger immunometabolic alterations underscored by lipid droplet biogenesis. Aging reduces the capacity to control these responses causing increased and persistent inflammation, metabolic dysregulation, and impaired cellular waste disposal. In turn, chronic peripheral inflammation during aging induces immunosenescence further worsening stroke-induced immunodepression, thus increasing the risk of post-stroke infection. Aging also alters gut microbiota composition inducing dysbiosis. These changes are enhanced by age-related diseases, such as atherosclerosis and type-II diabetes, that further promote vascular aging, predispose to stroke, and exacerbate brain inflammation after stroke. Current advances in aging research suggest that some age-associated alterations may be reversed. Future work will unravel whether such evolving anti-aging research may enable designing strategies to improve stroke outcome in the elderly.

## Introduction

Aging is associated to a certain degree with cognitive decline, and it is a critical risk factor for neurodegeneration [1]. The increase in life expectancy parallels the raise of age-dependent cognitive impairment and dementia associated to neurodegenerative and vascular conditions. Epidemiological data reveal that 84% of all stroke cases worldwide occur every year in people aged above 49 years [2]. Then, the risk of stroke increases every year of age by 9% in men and 10% in women [3], and, accordingly, stroke prevalence increases with age in both sexes [4]. Moreover, stroke has worse functional consequences in the elderly, as assessed with different prognostic scales [5]. It is estimated that stroke accelerates the age-dependent functional decline by nearly tripling the spontaneous annual increase in disability [4]. A critical reason underlying the worse response of the elderly to stroke is the age-related increase in frailty. The frailty status, as assessed with several indexes and scales, is common in stroke patients and it is related to poor outcomes [6]. Aging-associated frailty is due to the deterioration of tissue and organ functions, which enhances the risk of developing age-associated diseases. Frailty during aging is highly variable between individuals of the same age. Accordingly, individuals have a different capacity of brain resilience to cope with and respond to challenges such as stroke. Age has an impact on neuronal activity and viability, glial cell function, structure and function of brain blood vessels and the blood-brain barrier (BBB), and the bidirectional communication between the brain and the periphery. Given the effects of age in global functional decline, the age factor must be considered to understand stroke pathophysiology.

### Aging factors relevant for stroke outcome

**Fig. 1:**
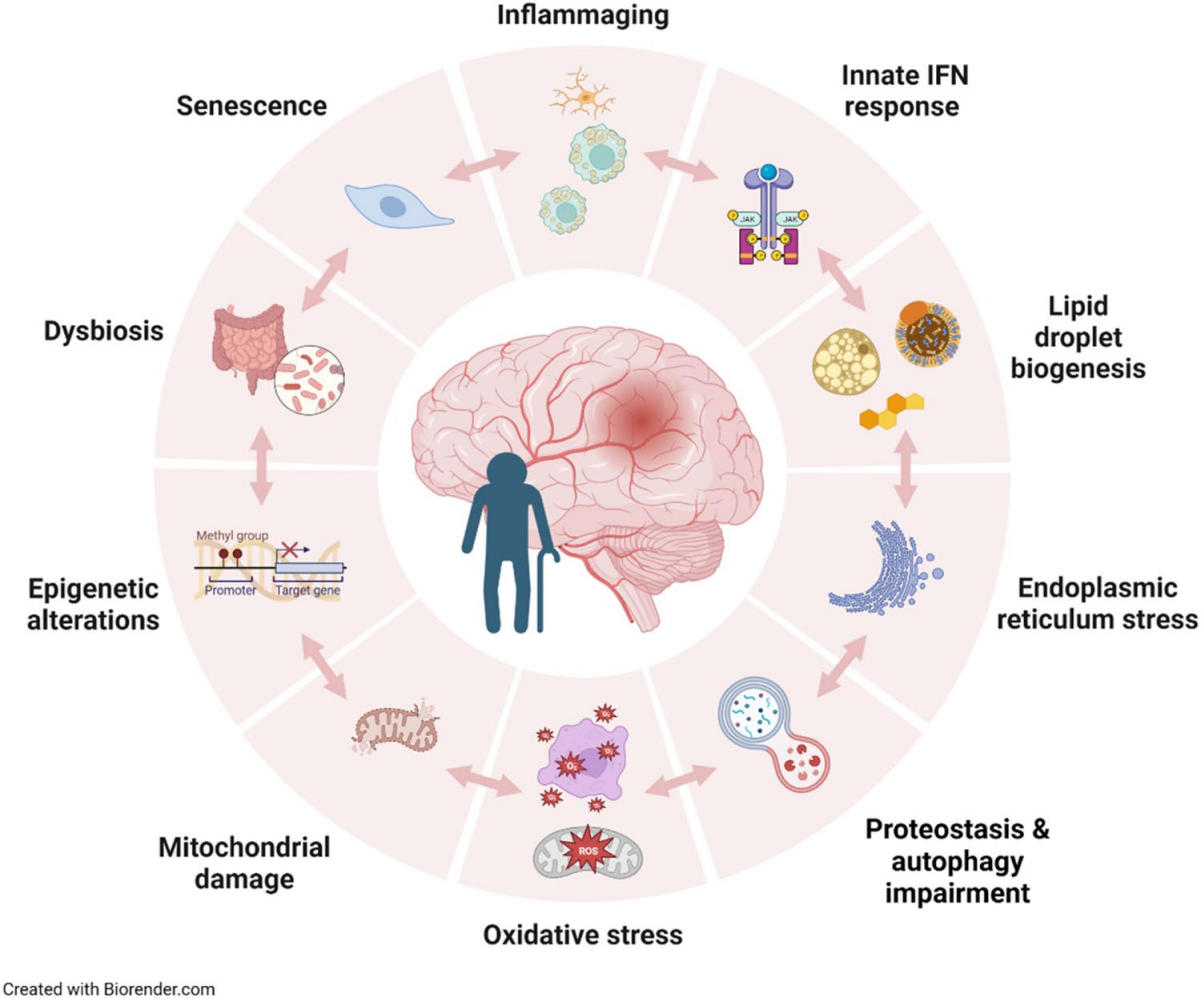
Chronic aging factors influencing stroke outcome in the elderly. Selection of aging hallmarks that are expected to affect stroke outcome converging through diverse and interrelated mechanisms, causing overactivation of innate immune responses in the brain. As a consequence, after stroke, the aged brain shows exaggerated production of inflammatory mediators and failure to resolve the inflammatory response, which persists for longer in the elderly

Complex and interrelated factors are associated to functional decline during aging. A seminal paper by López-Otín and co-workers [7] identified 9 hallmarks of aging, recently updated to 12 ones [8], namely genomic instability, telomere attrition, epigenetic alterations, loss of proteostasis, deregulated nutrient sensing, mitochondrial dysfunction, stem cell exhaustion, altered intercellular communication, cellular senescence, disabled macroautophagy, chronic inflammation, and dysbiosis. As in global aging, the same mechanisms listed above intervene in brain aging and cognitive decline, and they most likely play a role in stroke outcome in the elderly. Moreover, the chronic modifications progressively induced by aging may additively or even synergistically amplify the alterations induced by stroke and prolong the responses to acute brain damage. We will briefly discuss some of these aging factors for their putative involvement in stroke pathophysiology and will expand on the concept of chronic inflammation in the elderly (Fig. 1).

Aging promotes epigenetic changes mediated by DNA methylation, histone modification and expression of non-coding RNA. Notably, aging-induced changes in the DNA methylation pattern are used as epigenetic clocks to predict the ‘functional or biological age’, which is dependent on genetic and environmental factors, versus the ‘chronological age’ [9]. Epigenetic modifications occur in all cells, accumulate with age, and are affected by disease conditions and experiences during life. Stressful experiences and other environmental factors cumulatively promote epigenetic changes and somatic mutations that can accelerate the biological age [10]. Accordingly, the rate of functional decline is highly variable between individuals [11], and it is more related to the biological age rather than the chronological age. By similarity, the biological age rather than the chronological age is determinant in the worsening effect of age on stroke outcome [12].

Loss of proteostasis and disabled macroautophagy (termed autophagy in this review) may be particularly relevant at the cellular level in long lived brain cells. Alteration of these fundamental cellular processes is expected to impair cellular waste disposal leading to the accumulation of protein aggregates and lipids. Accordingly, an autofluorescent pigment termed lipofuscin, which is composed of residual lysosome products, can be typically seen by microscopy in cells of brain tissue sections from old individuals [13]. Dysfunction of autophagy is associated to a wide range of disease conditions, and it is a hallmark of aging [14]. Stroke perturbs autophagy by increasing the autophagic flux, but this phenomenon is associated with dysfunctional lysosomal storage and synaptic activity in neurons [15], and stroke-induced alterations in autophagy are associated with dysfunctional phagocytosis in microglia [16]. Dysregulation of autophagy after stroke is complex since it may have cell type–dependent consequences, and the response of the aged brain is expected to differ from the young. Moreover, alterations of autophagy and strongly related impairment of waste disposal are also interconnected with endoplasmic reticulum stress, oxidative stress and inflammation [17]. Other aging factors that may be important in stroke include mitochondrial dysfunction, as recently reviewed elsewhere [18], and age-dependent decrease in neurogenesis due to reduced neural stem cell proliferation, which in turn may depend on epigenetic deregulation [19]. Interestingly, the same processes progressively affected by aging suffer acute and strong perturbations after stroke. Moreover, several stroke-induced disturbances seem to resolve faster in young rather than old individuals.

Finally, aging harbours the accumulation of senescent cells, i.e. cells undergoing irreversible growth arrest and secreting pro-inflammatory cytokines and other molecules conforming the senescence-associated secretory phenotype (SASP) [20]. This phenotype is mainly detected in division-competent cells, such as stem cells. Senescence of stem cells has been related to age-dependent functional decline, for instance in the hematopoietic system [21]. SASP may also contribute to the status of low-grade chronic inflammation associated with aging and known as ‘inflammaging’.

### Inflammaging

Inflammaging involves a global upregulation of innate immune mediators in the elderly [22, 23]. Typical molecules associated to senescence-related inflammation are interleukin-6 (IL-6), tumor necrosis factor alpha (TNF-a), growth factors, acute-phase reactants, and other pro-inflammatory molecules, as well as autoantibodies. Importantly, inflammatory stimuli from the periphery may affect the brain, thus peripheral senescent cells may have an impact on brain inflammation. However, cellular senescence has also been related to tissue repair and has been proposed as a mechanism to alert other cells of functional alterations that can trigger a repair response [20].

In the brain, aging induces a specific transcriptomic signature highlighted by increased inflammation and microglial cell priming [24]. Stroke triggers an acute and sharp inflammatory response, both in the brain and the periphery, to set up mechanisms than can restore homeostasis. The microglial inflammatory response to stroke is exacerbated in the elderly [24, 25]. Microglia cells also display a more pro-inflammatory, dystrophic and dysfunctional phenotype in the elderly [26, 27]. It is likely that the higher production of inflammatory mediators by microglia in the aged brain may occur due to a deficient control of inflammation. Accordingly, the inflammatory response to acute stroke persists for longer in old rather than young mice [24]. Given the involvement of microglia in maintaining the integrity of the BBB, it has been suggested that changes in microglia of the aging brain contribute to the exacerbated BBB breakdown caused by hypoxia in the elderly [28].

### Age and stroke-induced innate immune responses

Following stroke, injured cells generate danger signals or damage-associated molecular patterns, including nuclear proteins, nucleic acids, heat-shock proteins, amongst others, triggering immune responses by activating pattern recognition receptors (PRRs) [29]. Microglial cells are equipped with membrane PRRs enabling sensing danger signals from the environment, and intracellular PRRs that recognize cytosolic nucleic acids resulting from viral infection or cellular stress and damage. For instance, stroke activates the cyclic GMP-AMP synthase (cGAS) pathway, which induces inflammation and brain damage [30, 31]. cGAS senses cytosolic double-stranded DNA, e.g. mitochondrial DNA (mtDNA) leaked after mitochondrial damage, and activates the receptor stimulator of interferon (STING) inducing the type I interferon (IFN) response [32]. Type I IFNs (α, β, and ω) bind to IFN receptor type 1, and they signal through JAK1 and TYK2, phosphorylating STAT1, STAT2, and STAT3, as well as other STAT family members. Type I IFN-stimulated genes (ISG) are involved in antiviral defence through induction of innate immune responses and antiproliferative activities, amongst others [33]. This pathway must be precisely regulated given that gain of function of STING and subsequent chronic IFN type I overproduction is involved in the pathogenesis of autoimmune diseases [34].

Stroke induces a strong activation of the type I IFN program in microglia [35, 36]. The microglial IFN response is superior to that detected in brain infiltrating immune cells like dendritic cells [36], suggesting that microglia are particularly prone to deploy the transcription of ISGs after stroke. In the aged brain, stroke further exaggerates the magnitude of the type I IFN response in microglia and oligodendrocytes [24, 25]. Furthermore, the microglial IFN response to stroke persists for longer in aged mice compared with young mice [24]. Overactivation of the IFN program in the aging brain could be mediated by senescent cells since they generate cytosolic chromatin fragments that are recognized by cGAS, inducing the activation of STING and the production of SASP factors [37]. Notably, premature aging in the Hutchinson-Gilford progeria syndrome (HGPS) is associated to a potent STAT1-mediated IFN response that appears to be involved in cellular decline [38].

The microglia type I IFN program mediated by Stat1 transcription factor intervenes in immunometabolic changes involving the accumulation of lipid storage organelles in the cytoplasm called lipid droplets [25], which are functional metabolic hubs and innate immunity first responders [39]. After stroke, the proportion of microglia with lipid droplets increased 8.3-fold compared to sham-operated mice, suggesting that this effect is part of an adaptation to the immunometabolic challenge imposed by stroke. Lipid droplets were described in microglia of old mice under steady state accompanied by increased oxidative stress and an inflammatory phenotype [40]. Accordingly, the proportion of lipid-droplet rich microglia increased from less than 3% in young mice to nearly 14% in 21–22-month-old mice under control conditions (sham-operation) [25]. Lipid droplet-rich microglia in old mice further increased by 2.7-fold after ischemia [25]. This effect suggests an attenuated acute adaptive reaction to the stroke challenge in old compared to young microglia. Lipid droplet accumulation in old microglia may be part of the primed phenotype observed in microglial cells of the aged brain that acquire some features of disease-associated microglia (DAM) [41, 42]. The white matter of aged mice shows degenerating myelin and associated microglia display overrepresentation of genes involved in phagocytic activity and lipid metabolism likely related to myelin removal [43]. Overload of phagocytosed lipid-rich material under natural aging or after disease conditions may surpass the cellular lipid disposal capacity promoting metabolic adaptations and lipid accumulation [44]. Independently, inflammation may also trigger acute lipid droplet biogenesis, as shown in microglia cell cultures treated with lipopolysaccharide [40, 45] or in acute stroke [25]. These findings suggest that diverse signals converging in immunometabolic alterations result in the formation of lipid droplets in microglia.

Overall, stroke induces an acute transcriptional and immunometabolic program in microglia of young mice with features resembling some of the phenotypic changes displayed by microglia of aged mice under steady state. Stroke in aged mice further exacerbates this response triggering more inflammation and failing to exert an adequate regulatory control to terminate the response to the acute challenge.

### Immunosenescence

Age-dependent inflammation is strongly associated with defects in the immune system. The capacity to mount adaptive and innate immune responses is attenuated in the elderly due to immunosenescence that explains why old people have an increased susceptibility to infections. This immunosuppressive state generated in the elderly is most likely a counteractive response trying to restrain the chronic inflammation. Decrease in naïve T cells and increase in memory T cells as well as lower capacity to generate antibody reactions against pathogens are prominent features of immunosenescence [46]. In fact, the immunosenescent state is characterized by a dysfunctional activity in almost all immune cell types and an increase in the activity of suppressor cells, including regulatory T cells, myeloid-derived suppressor cells, and regulatory B cells [47]. Older people retain pathogen-specific immune memory obtained when young. However, their response to new infections is often low, in part because of the malfunctioning of innate immune cells. Indeed, the capacity of macrophages and neutrophils to react against a stimulus and exert effector functions is reduced in the elderly. For example, macrophage activation is impaired in older mice in addition to showing a reduced phagocytic activity and limited production of superoxide and nitric oxide [48]. Dendritic cells show a reduced migration capacity to the lymph nodes and express less co-stimulatory markers, impairing their function as antigen-presenting cells [49]. Natural Killer cells have a limited production of cytotoxic granules too [50]. Neutrophils of old individuals show a reduction in superoxide and chemotaxin production that ends up in a declined bactericidal activity [51].

The acute brain inflammation induced by stroke is followed by systemic immunodepression [52–55]. The combination of aging-derived immunosenescence with post-stroke immunodepression will further increase the probability of developing infections in older stroke sufferers. Therefore, improving the function of the immune system in the elderly will surely help to limit this very important post-stroke complication. Overall, the immunological alterations induced by aging appear to be critically involved in the worse outcome of stroke in the elderly. Importantly, several lines of evidence suggest that the peripheral immune system and blood factors of the elderly contribute to age-dependent cognitive decline [56, 57] and exacerbate the stroke brain lesion [58].

### Vascular aging

Blood factors and immune cells may influence stroke outcome by affecting the function of brain vessels. The status of the vasculature is a critical player in the individual response to aging, as postulated long ago by the English physician Thomas Sydenham in the seventeenth century, who wrote the famous quote ‘A man is as old as his arteries’. All aging factors described above affect the vasculature, as recently reviewed [59]. The aging brain vessels develop characteristic features of vascular dysfunction including increased BBB permeability, rarefaction, and formation of string vessels [60]. Loss of BBB integrity can facilitate access of blood molecules to the brain inducing inflammation, and in turn, inflammation may weaken the BBB integrity. Increases in BBB permeability are detected in healthy aging, and this phenomenon is further exacerbated in patients with vascular or Alzheimer’s dementia [61]. Accordingly, the brain blood vessels are more prone to rupture in the elderly increasing the rate of hemorrhagic transformation after ischemic stroke [58]. Age-associated BBB dysfunction may have secondary effects, like the induction of transforming growth factor-β (TGFβ) in astrocytes impairing neuronal function associated with age-dependent functional decline [62]. Further studies will determine whether and which vascular factors and components of the peripheral immune system in the elderly may contribute to the described spontaneous age-dependent leakage of the BBB and promote subsequent brain inflammation exacerbating stroke brain damage.

### Microbiota

The intestinal microbiota has been implicated in normal development of the brain, including the normal functioning of microglia [63] and the development of the BBB [64], but it has been also associated to multiple brain diseases, including stroke [65–67]. Stroke pathophysiology is affected by the intestinal microbiota [65–70] (see review [71]), and the intestinal microbiota is disrupted by stroke [67, 72, 73]. Transplantation of microbiota of mice with stroke into naïve germ-free mice followed by induction of stroke in these animals increased the size of the brain lesion, suggesting that stroke might induce a pro-inflammatory bias in the intestinal microbiota [67, 68]. Since it has been demonstrated that the intestinal immune status can be translated into the central nervous system (specifically, into the meningeal compartment through cell migration of IL17γδ-T cells from the small intestine to the meninges), stroke neuroprotection can be achieved by remodeling the intestinal immune system to a more “anti-inflammatory” phenotype, consisting of an increase in regulatory T cells and a reduction in IL17 producing γδ-T cells (IL17 γδ-T cells) [65]. After stroke, the immune status in the gut was translated into the central nervous system, specifically, into the meningeal compartment through cell migration of IL17γδ-T cells from the small intestine to the meninges. Similar findings were furtherly obtained when using different antibiotic cocktails to induce intestinal dysbiosis [66]. Noteworthy, other studies using broad-spectrum antibiotic cocktails failed to induce any protection from brain damage after stroke [69]. Intestinal microbiota alterations are highly depend on the type of antibiotics and also on the original composition of the microbiota that highly depends on the breeding conditions and even on the commercial breeders that provide the animal models [70].

Stroke-induced dysbiosis can secondarily affect the stroke outcome by a feedback loop between brain-gut-brain bidirectional pathway. Therefore, any process that alters the intestinal microbiota is susceptible of affecting stroke outcome. In this sense several vascular risk factors have been associated to intestinal dysbiosis, including diabetes, obesity, or hypertension, as well as aging [74]. Aging induces changes in the microbiota composition and, at the same time, alterations in the microbiome affect the rate of age-related decline. Some microbial commensals are lost during aging (e.g. Bifidobacterium), some commensals increase (e.g. Akkermansia) and some pathobionts are over-represented (e.g. Enterobacteriaceae) [75]. Whether these changes are consequence of general physiological decline, including inflammaging is still an open question. Of note, acute stroke causes a bloom of Enterobacteriaceae in the gut microbiota [76], that in the elderly will add to their basal increase in opportunistic commensals. Aging effects on microbiota, through their impact on the immune homeostasis, will plausibly affect stroke pathophysiology in old individuals and promote a poorer outcome. In addition, changes in commensal/pathobiont derived metabolites have been also reported. Thus, aging is associated with an increase in the production of detrimental metabolites and the consumption of beneficial metabolites [77]. For example, butyrate, a short-chain fatty acid (SCFA) metabolite that has been associated with a healthy status of microbiota is reduced with aging [78] and, this reduction is less severe in healthy centenarians [79]. Moreover, acute reduction of fecal SCFA is reported in stroke patients [80]. Therefore, both aging and stroke affect the intestinal microbiota shifting the intestine to a more pro-inflammatory state.

### Co-morbidities

Several additional co-morbidities that are commonly present in stroke patients further increase systemic inflammation. We will focus on two of the most important ones: atherosclerosis and diabetes. Overall, a common factor amongst aging, stroke, atherosclerosis and type II diabetes is the contribution of chronic inflammation to the pathophysiological process. Controlling chronic inflammation should have a positive impact on the aging process, the progression of important comorbidities, and the outcome of stroke in the elderly.

#### Atherosclerosis

Atherosclerosis is a chronic inflammatory condition more prevalent in the elderly and therefore associated with aging. Atherosclerosis increases the risk of stroke, and in turn stroke increases the progression of atherosclerosis. Both directions are mediated, at least in part, by systemic inflammation. Inflammation has a fundamental role in every step of atherosclerosis formation, from the initial points to the final complications of thrombosis. Atherosclerosis is a hardening and narrowing of arteries caused specially by the accumulation of cholesterol plaques in the inner lining of an artery, inducing inflammatory mediators, such as IL-6, and the recruitment of leukocytes to the arterial wall [81]. Increase in IL-6 affects hematopoietic stem cells in the bone marrow promoting the production of myeloid cells with a higher capacity to produce inflammatory cytokines such as IL-6 and IL-1β, thus entering in a positive feedback loop of re-inflammation [82]. Inflammation and matrix remodeling facilitate atheroma plaque destabilization and rupture that may lead to stroke [83]. In turn, the acute inflammatory response caused by stroke will contribute even further to the progression of the atherosclerosis in the whole organism [84]. Atherosclerosis, aging, and stroke seem to be a triad of factors that reinforced each other in a process strongly linked to inflammation. Therefore, efficient managing of chronic inflammation during the progression of atherosclerosis/aging and post-stroke inflammatory responses may have a beneficial effect to prevent stroke and its secondary complications.

#### Type II diabetes

The risk of type II diabetes increases with age, affecting around 25% of the population over 65 years old. Aging is associated with an increase in abdominal obesity, a major contributor of insulin resistance, and therefore aging and type II diabetes are intricately linked. In addition, aging affects adipose tissue homeostasis and metabolic functions both of which decline with aging and obesity. Aging is associated to adipose tissue senescence, which causes defective adipogenesis, inflammation and insulin resistance [85]. Patients with diabetes present more than double the risk of stroke and, diabetes and/or high glucose levels at the onset of stroke have been associated with worse outcomes [86], related at least in part to glucose-driven oxidative stress [87]. Type II diabetes influences stroke outcome in several different ways. First, hyperglycemia induces brain infarct growth [86]. Second, hyperglycemia primes the thromboinflammatory cascade by activating the endothelium, platelets and neutrophils [86]. Third, diabetic patients show increased susceptibility to infections [88]. Fourth, diabetes also promotes atherogenesis [89]. Finally, type II diabetes, triggered by insulin resistance is caused by a mechanism involving chronic inflammation. As with other stroke comorbidities, ameliorating age-associated diabetes may have an impact not only reducing stroke incidence but also improving stroke prognosis.

### Future therapeutic options

Finding ways to prevent aging has been for centuries the search for the Holy Grail. However, recent solid findings suggest that some hallmarks of aging may be reversible. For instance, loss of epigenetic information is postulated as a mechanism causative of aging that can be reversed [90]. Indeed, interventions on the aging epigenetic landscape to attempt its rejuvenation emerge as putative strategies to delay aging or promote healthy aging [91]. An astonishing finding in the field of brain aging was the rejuvenating effect of providing blood of young mice to old mice [56]. The study used parabiotic mice and the findings pointed to some soluble blood factor able to communicate with the brain to improve cognitive functions that had deteriorated due to aging, thus implying that the cognitive decline may be reversible, at least in part. Recent studies also suggest that immunosenescence can be modified since transplantation of splenocytes from old mice to young mice caused immunosenescence in the latter, while this phenomenon was attenuated by transplantation of young splenocytes to old mice [57]. Several lines of evidence suggest that improving aging features has an impact in stroke outcome too. Rietzel and co-workers [58] performed interesting experiments using transplantation of bone marrow of young mice to old mice reporting improvement of several signs of brain aging, such as reduction in certain growth factors and improved behavioural performance of some motor tasks. Notably, induction of stroke in these heterochronic old mice with young bone marrow resulted in milder behavioural deficits compared with those in corresponding controls [58].

Other studies also suggested that age-dependent BBB dysfunction might be attenuated or reversed. For instance, inhibition of TGF-β diminished neuronal dysfunction mediated by age-associated BBB alteration [62]. Long-term treatment of old mice with small extracellular vesicles derived from inducible pluripotent stem cells (iPSCs) attenuated signs of BBB senescence and, after stroke, treated mice showed protection of BBB integrity, attenuated inflammatory responses, showed less neuronal death and better neurofunctional recovery [92]. Age-dependent impairment of the autophagic flux has been improved by dietary administration of spermidine, a polyamine that naturally induces autophagy [93]. Nevertheless, stroke increases the autophagic flux and there are controversial results in the field regarding the effect of drugs acting on autophagy. Pharmacological fine tuning of the process may be the key to regulate autophagy after stroke [94]. However, the age factor must be considered since the marked effects of aging may be critical in the response of the processes of autophagy to stroke. Drugs called senolytics can eliminate senescent cells and improve the aging phenotype [95, 96]. Targeting senescent cells can attenuate age-dependent cognitive decline, at least in animals [97]. Moreover, several lines of evidence suggest that eliminating senescent cells may also have therapeutic value in the treatment of ischemic stroke [98, 99].

Interventions directed to influence the microbiota composition and derived metabolites may offer new therapeutic opportunities for implementing the treatment of stroke in the elderly. Notably, transplantation of fecal matter from young mice enriched in butyrate-producing bacteria to old mice can reverse at least part of the phenotypes associated with aging by improving cognitive functions [100]. Furthermore, a seminal study demonstrated that the gut microbiota composition of uninjured old mice was similar to that altered after stroke in young mice [78]. Then, microbiota manipulation by fecal transplants showed that young mice harboring an “old-microbiota” developed higher deficits post-stroke and that aged mice with “young-microbiota” developed milder post-stroke alterations. The latter phenotype was also accompanied by a reduction in post-stroke mortality and circulating immune markers.

## Final remarks

Aging is associated with a plethora of alterations that increase frailty, predispose to age-related diseases, and reduce the brain resilience to injury (Fig. 1). Not only stroke risk increases with age, but stroke outcome is worse in the elderly. Critical players are aging-induced intrinsic alterations of brain cell function, the BBB, and the immune system. Two main hallmarks of aging, namely chronic age-associated inflammation or inflammaging and immunosenescence, impact the outcome of stroke. The acute inflammatory and immunometabolic reactions triggered by stroke appear to be exacerbated in the elderly and, perhaps more importantly, they fail to resolve and persist for longer in aged compared with young individuals. Moreover, the combination of peripheral inflammation and the more immunosuppressive status of the elderly will facilitate stroke-associated immunodepression and increase the risk of post-stroke infection. Possibly, future drugs or interventions that promise to slow down or even reverse some aging features may also delay or reduce the rate of cognitive decline and onset of age-associated diseases, including stroke. Moreover, in the event of stroke, such treatments may promote a better functional outcome. Future studies will determine whether drugs targeting aging-related features may also be a therapeutic option in acute stroke capable of improving stroke outcome in the elderly.
